# P-773. Impact of COVID-19 Pandemic Clinical Curtailments on Urine Microbial Diversity

**DOI:** 10.1093/ofid/ofaf695.984

**Published:** 2026-01-11

**Authors:** Luis F Barroso, Michael E DeWitt, Brinkley R Bellotti, Jennifer J Wenner, John W Sanders, Charles A de Comarmond

**Affiliations:** Wake Forest University School of Medicine, Winston Salem, North Carolina; Atrium Wake Forest Baptist Health/ Wake Forest University School of Medicine, Winston-Salem, North Carolina; Wake Forest University School of Medicine, Winston Salem, North Carolina; Wake Forest University School of Medicine, Winston Salem, North Carolina; Wake Forest University School of Medicine, Winston Salem, North Carolina; WG "Bill" Hefner VAMC, Salisbury, North Carolina

## Abstract

**Background:**

Clinicians often obtain urine analysis (UA) for many indications. Healthcare systems often bundle reflex urine cultures to abnormal urine analysis to avoid the need for recollection of urine specimen. In the aging Veterans Affairs (VA) population with relatively large number of patients with chronic bladder colonization or asymptomatic bacteriuria (ASB), such reflex testing can result in unnecessary exposure to antibiotics. Lockdown during the COVID-19 pandemic resulted in fewer patients obtaining medical testing resulting in decreased UAs and cultures in asymptomatic patients. This study evaluated urine microbial diversity and the rates of antimicrobial therapy in patients with ASB before and during the COVID-19 at a single VA facility.

**Figure 1:**
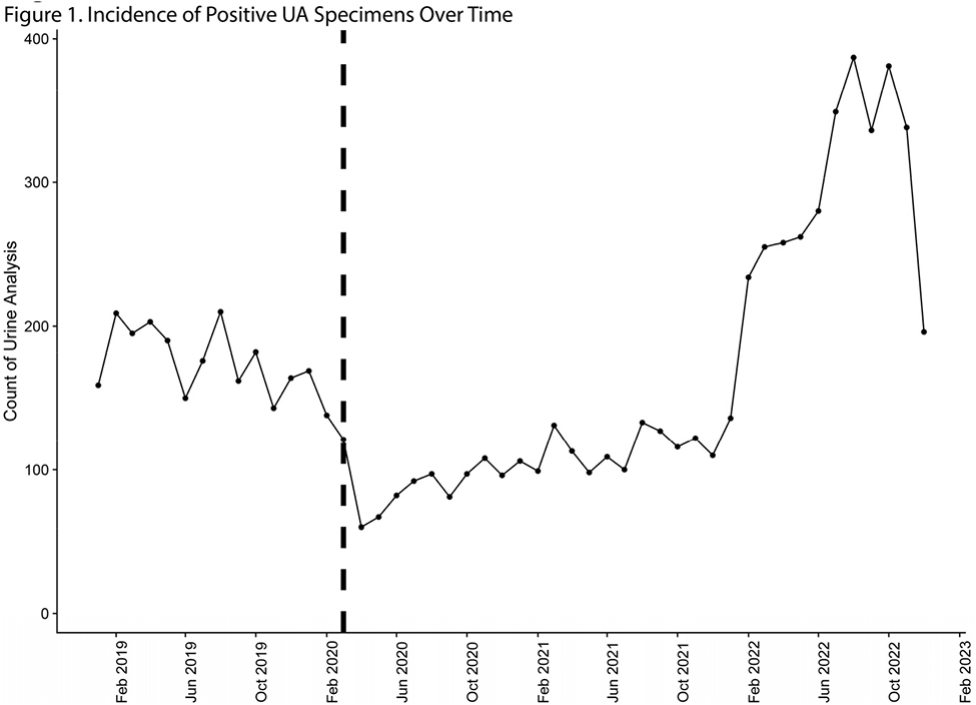
Incidence of Positive UA Specimens Over Time

**Figure 2:**
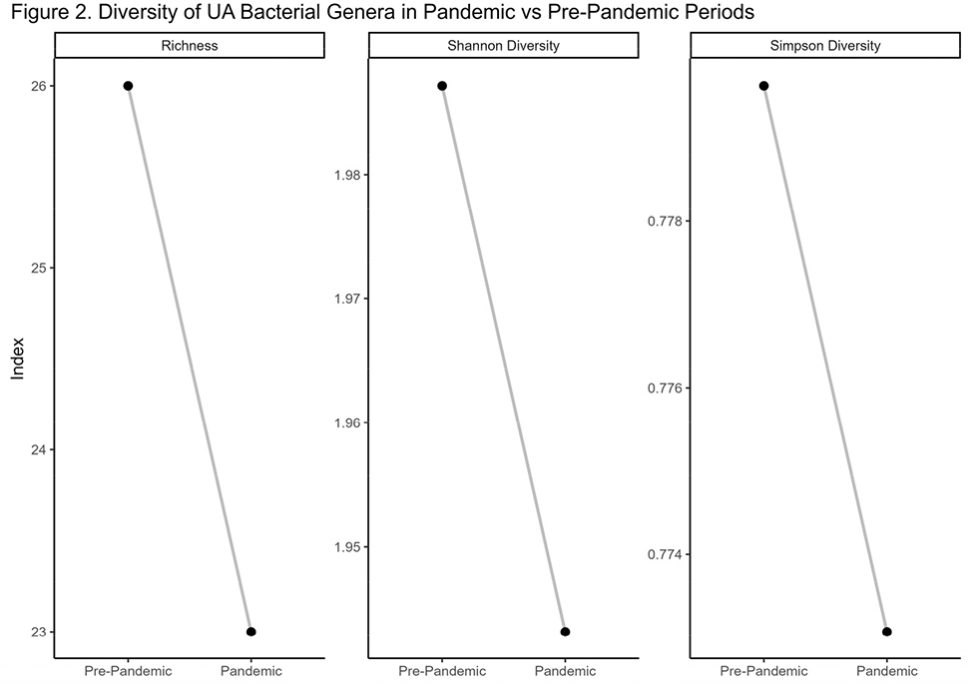
Diversity of UA Bacterial Genera in Pandemic vs Pre-Pandemic Periods

**Methods:**

We collected all positive UA results from Jan 1, 2019 to December 1, 2022. Patient demographics, UA results, and treatments were collected. Descriptive and inferential statistics were performed and indexes of diversity including richness, Simpson diversity and Shannon diversity were calculated. Prevalence ratios were calculated with Poisson regression and robust standard errors. The pandemic period was considered any date after March 1, 2020.

**Figure 3:**
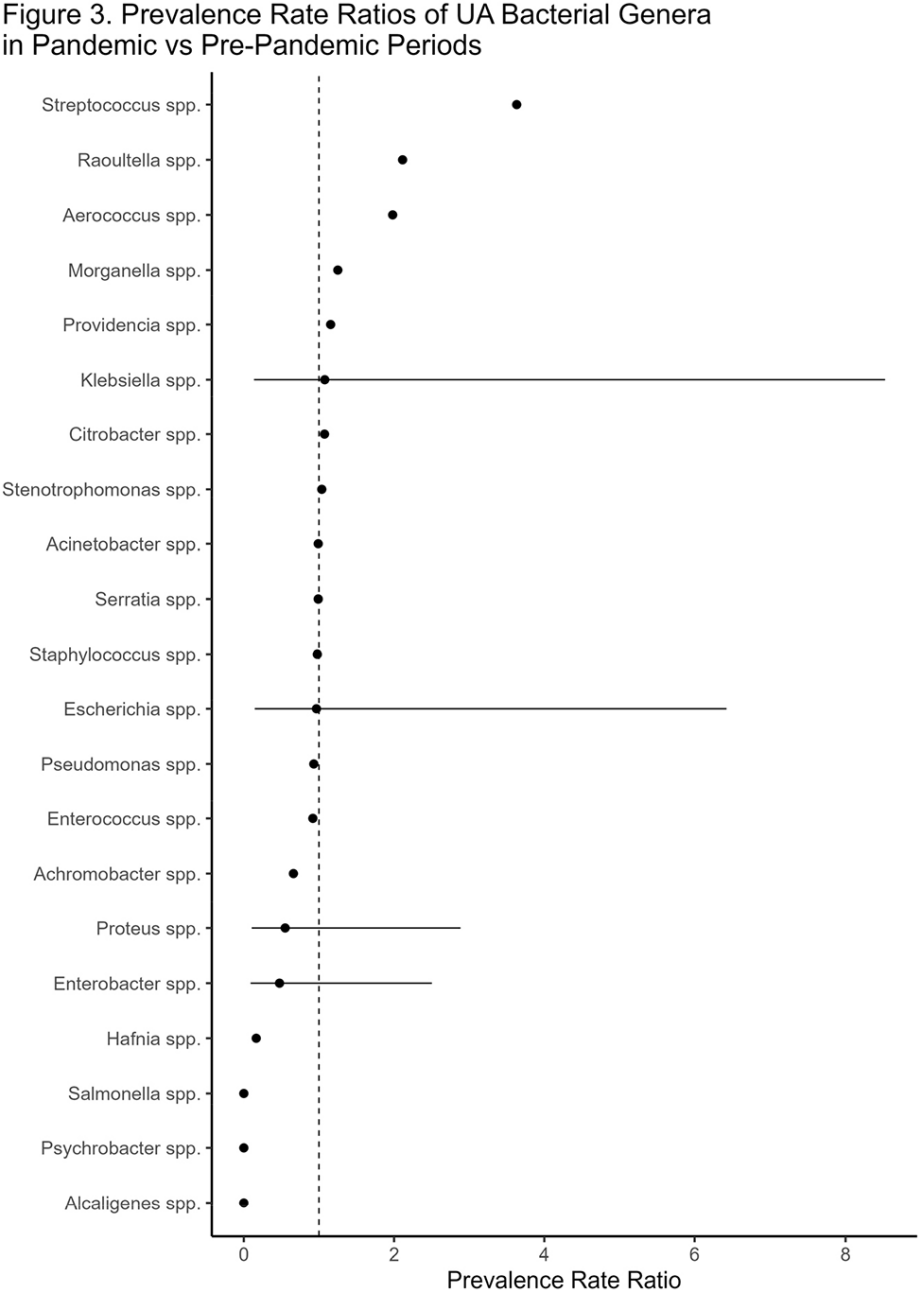
Prevalence Rate Ratios of UA Bacterial Genera in Pandemic vs Pre-Pandemic Periods

**Results:**

A total of 8,127 specimens were collected with 2,450 in the pre-pandemic period and 5,677 in the pandemic period (Figure 1). Males were more likely to have a positive UA (p < 0.001) and symptomatic UAs were more common (p< 0.001) in the pandemic period. The proportion of extended-spectrum beta-lactamase producing cultures was identical (4.5%, p > 0.9). All measures of microbial diversity decreased during the pandemic period (Figure 2). The prevalence rate ratio doubled in *Streptococcus*, *Raoultella*, and *Aerococcus* spps, while the prevalence rate ratio more than halved for *Hafnia*, *Psychobacter*, *Alcaligenes* spps (Figure 3).

**Conclusion:**

Decreased UA and culture sampling from asymptomatic patients resulted in different microbiologic profiles in culture results. Decreased microbial diversity suggests that symptomatic patients have a narrower range of pathogens that cause true infection.

**Disclosures:**

Luis F. Barroso, MD, UpToDate: Advisor/Consultant

